# Visual analytics and rendering for tunnel crack analysis: A methodological approach for integrating geometric and attribute data

**DOI:** 10.1007/s00371-016-1257-5

**Published:** 2016-05-11

**Authors:** Thomas Ortner, Johannes Sorger, Harald Piringer, Gerd Hesina, Eduard Gröller

**Affiliations:** 1 VRVis Research Center, Vienna, Austria; 2 TU Wien, Vienna, Austria

**Keywords:** 3D real-time rendering, Visual analytics, Integration of spatial and non-spatial data, Methodology

## Abstract

The visual analysis of surface cracks plays an essential role in tunnel maintenance when assessing the condition of a tunnel. To identify patterns of cracks, which endanger the structural integrity of its concrete surface, analysts need an integrated solution for visual analysis of geometric and multivariate data to decide if issuing a repair project is necessary. The primary contribution of this work is a design study, supporting tunnel crack analysis by tightly integrating geometric and attribute views to allow users a holistic visual analysis of geometric representations and multivariate attributes. Our secondary contribution is Visual Analytics and Rendering, a methodological approach which addresses challenges and recurring design questions in integrated systems. We evaluated the tunnel crack analysis solution in informal feedback sessions with experts from tunnel maintenance and surveying. We substantiated the derived methodology by providing guidelines and linking it to examples from the literature.

**Electronic supplementary material:**

The online version of this article (doi:10.1007/s00371-016-1257-5) contains supplementary material, which is available to authorized users.

## Introduction

The detection and documentation of cracks in the concrete surface of a tunnel are essential for assessing its condition. These cracks comprise a 3D polyline and several multivariate attribute values, such as length, width, orientation, and moisture. Tasks of analysts are, for instance, to identify patterns which endanger the structural integrity of the tunnel surface or assess the density of cracks along the tunnel and identify critical sections. Accomplishing such tasks and evaluating if a repair project is necessary typically requires the visual analysis of detailed geometric data and multivariate attributes simultaneously.

The historical workflow in tunnel maintenance involves an analyst inspecting the tunnel surface on-site. Meanwhile inspections are mostly performed virtually on detailed, digitally reconstructed 3D models of the tunnel surface. Tunnel cracks are traced in high-resolution images by semi-automatic crack-detection algorithms. However, the analysis of multivariate data is still mostly performed via spreadsheets and static plots. Since geometric and attribute data are evaluated separately, no integrated workflow is supported resulting in tedious work to relate both aspects of the data.

### Contributions

The primary contribution of this paper is a design study for visual analysis of tunnel cracks in the context of tunnel maintenance. Abstracting from the specific problem domain of tunnel crack analysis we identified a general problem space emerging from the combination of geometric and attribute views, including obstacles and recurring design questions. Therefore, as a secondary contribution, we present VISAR (Visual Analytics and Rendering), a methodology to support system designers in creating integrated solutions that combine geometric and attribute data.

## Related work

A vast number of commercial and research-based tools for multivariate analysis exist, which allow users to explore data from different points of view [15, 18, 29, 32, 40]. Roberts [26] provides an extensive survey on coordinated multiple view (CMV) systems, which typically do not offer geometric rendering. 3D rendering engines are capable of rendering large sets of geometric data at interactive frame rates, but do not provide analytical capabilities. Widespread in engineering and urban planning are geographic information systems (GIS) or cartographic visualizations. These applications form a compromise between detailed spatial rendering and multivariate analysis, but typically either lack complex analytical capabilities [9] or are restricted to cartographic views [15].

Solutions providing sufficiently detailed geometric rendering and means for the analysis of multivariate attributes are often tailored to a specific use case, such as in disaster management [25], 3D visibility analysis [11, 22], urban data analysis [7], or lighting design [30]. These works combine visualizations of multivariate attributes with a detailed 3D spatial visualization for supporting decision-making in heterogeneous scenarios. Regardless of the problem domain, there are recurring challenges and design questions inherent to these heterogeneous scenarios, such as localization of an object in 3D space or identifying patterns across data domains. Although many authors use similar integration patterns, their discussion is often focused on a particular application rather than being generalized.

In the context of CMV, multiple models and frameworks have emerged. Wang Baldonado et al. [39] formulate several guidelines on when and how to use CMV. Boukhelifa et al. [3] present a model on how coordination can be formalized and implemented, while Sorger et al. [31] developed a taxonomy to classify integration techniques of spatial and attribute visualizations. Actual frameworks include Snap-Together [20] and Improvise [40], which are highly focused on the design and coordination of multiple views. However, neither of these frameworks deal with the special intricacies of integrating spatial geometric views and multivariate attribute views.

**Fig. 1:**
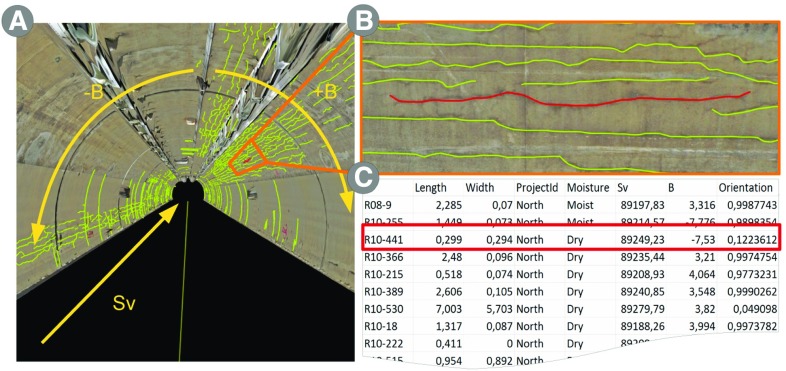
**a** Reconstructed mesh of the tunnel surface. Tunnels provide an additional metric coordinate system consisting of the value Sv running along the tunnels axis and B running perpendicular to it. Each crack comprises **b** its geometric representation, a 3D polyline, and **c** its attributes, such as length, width, moisture, and orientation. Red highlighting indicates the same crack across **a**, **b**, and **c**.

## Problem abstraction

In the following section, we provide details on a tunnel maintenance scenario and the intricacies of tunnel crack data. We follow with a discussion of the problem space of combining geometric and attribute views and identify potential obstacles impeding an interactive visual analysis of the surface cracks.

### Tunnel maintenance and tunnel crack analysis

A tunnel goes through three phases, planning, construction, and maintenance. An essential part of its maintenance is the documentation and evaluation of the development of cracks in its concrete surface. Based on these evaluations, maintenance projects are issued to repair or completely replace parts of the concrete surface. Tunnel surveying companies increasingly perform virtual inspections on reconstructed 3D tunnel models instead of doing on-site inspections. This minimizes downtimes of tunnels and enables a more comprehensible analysis. The surface data are acquired by a rail-mounted laser scanner platform in very high geometric resolution. At the same time, images are taken by high-resolution cameras. Co-registration of geometry and images results in a 3D textured mesh (Fig 1a). Ortner et al. [21] provide more detail on how the data are acquired, processed, and rendered. To extract the cracks, the image data are processed by a semi-automatic crack detection algorithm [23].

*Data abstraction* The center of our design study is the visualization of and the interaction with tunnel cracks. The course of each tunnel crack on the tunnel surface is described by a 3D polyline as geometric representation (Fig. 1b). Along with the geometry, each crack is associated with an attribute vector, consisting of the attributes *length, width, moisture, orientation* (Fig. 1c), and the additional coordinates *Sv* and *B*, which we discuss in the following paragraph. Moisture occurs in three categories, *dry, moist, wet* while orientation describes a crack’s angle in relation to the course of the tunnel. Its values range from $0^{\circ }$ to $90^{\circ }$, where $0^{\circ }$ corresponds to cracks aligned with the tunnel direction.

*The Sv/B coordinate system* Experts dealing with linear infrastructures often use a one-dimensional metric value to describe a position along a highway, railroad track, river, or tunnel. This value is often called stationing. In our case, the coordinate *Sv* describes meters along the tunnel. With another coordinate *B* running on the surface and being orthogonal to the axis, the surface position of every crack can be determined (Fig. 1a).

*Tunnel sections* Modern tunnel surfaces consist of spray concrete, which is wet concrete shot onto the raw excavation of the tunnel, resulting in a smooth surface. The concrete is applied continuously for a certain section of the tunnel, e.g., 120 m along the Sv coordinate, which forms a homogeneous surface. Individual cracks can be fixed by local injection of concrete, but larger damage typically requires the replacement of the concrete shell of a whole section. Therefore, it is essential for our users to investigate the number of cracks with respect to the Sv coordinate and identify potentially critical sections.

### Task analysis

Cooperating with experts from tunnel maintenance and tunnel surveying, we identified a series of tasks in the context of tunnel crack analysis.

- Identification of cracks with anomalous values regarding moisture, length, and orientation.
- Analysis of moisture distribution in the tunnel.
- Identification of critical sections based on the density of cracks.
- Identifying intersecting cracks with high moisture that endanger the structural integrity of the tunnel surface.
- Investigation if moisture in one tunnel tube is also present in an adjacent tube.

### Problem space of combining geometric and attribute views

Tory and Möller [33] distinguish between data types where the spatialization is given and where it is chosen. To visualize each facet of our data effectively, we decided to visualize the geometric representation in a 3D real-time rendering view, which we refer to as the *geometric view*. The attributes of each crack are visualized in views with chosen spatialization, such as scatter plots, parallel coordinates, and histograms, which we denote as *attribute views*. Coordinating these views by linking & brushing already allows analysts to utilize interactive visual analysis and discover phenomena that may not be apparent in a single view visualization [17].

**Fig. 2:**
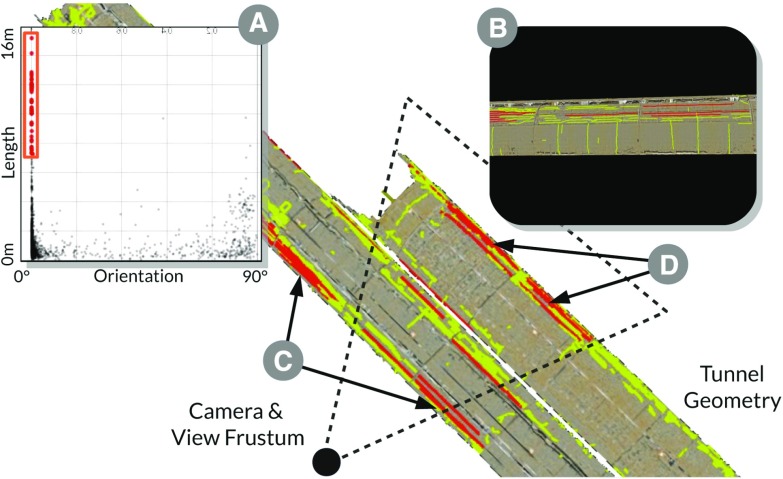
**a** 2D scatter plot showing orientation vs. length: linked selection of cracks, which are oriented along the tunnel direction, i.e., $0^{\circ }$, and which are longer than 8 m. **b** The scene rendered from the current camera position showing only some of the selected cracks (*red*). **c** Some are partially outside, others are completely outside the view frustum. **d** Cracks occluded by the first tunnel wall are not visible from the current camera position.

**Fig. 3:**
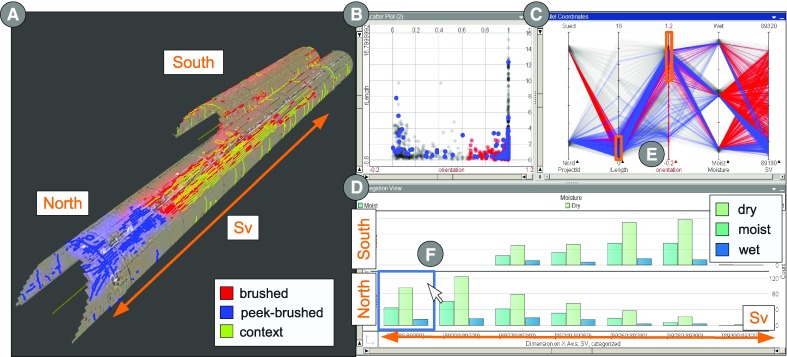
System overview: **a** the geometric view shows a textured mesh of north and south tunnel tube including tunnel cracks as polylines. **b** The scatter plot shows cracks as dots with respect to orientation on the *x*-axis vs. length on the *y*-axis. **c** The parallel coordinates plot enables the comparison and identification of trends for many dimensions. **d** The aggregation plot shows the distributions of moisture values in the north and south tunnel tube. Histograms are grouped to 120-m-long intervals of Sv. **e** Selections on length and orientation axis combined by logical AND. **f** Peek-brushing over the histogram causes blue highlights on the corresponding cracks in **a**, **b**, and **c**

However, the coordination of geometric and attribute views has numerous recurring challenges. Figure 2a shows an approach for enabling analysts to brush certain cracks in a scatter plot. To judge the spatial distribution, they need to identify the corresponding geometric representations in the geometric view. Some of these cracks may fully or partially lie outside (Fig. 2c) the view frustum of the geometric view (Fig. 2b), or they may be fully or partially occluded (Fig. 2d) by other geometric objects in the scene.

Elmqvist and Tsigas [8] define three *visual perception tasks*. In this context, we denote brushed cracks as part of the *focus* while the remaining cracks and the tunnel geometry are part of the *context* [14]. Applying their abstraction to our use case, the visual perception tasks can be described as follows:

- *Discovery* concerns locating a crack in the geometric view after it has been brushed in an attribute view.
- *Access* is concerned with retrieving the shape or color of a crack in the geometric view.
- *Spatial relation* concerns the assessment of spatial or geometric properties of brushed cracks with respect to other cracks or the tunnel surface geometry.

### Design goals

The situation depicted in Fig. 2 illustrates that cracks in focus can easily fail the visual perception tasks, due to occlusion or being positioned outside the view frustum. Our visual analysis tool must integrate geometric and attribute views as such that users are able to perform their specific tasks, while preventing the visual perception tasks from failing. Based on this conclusion, we derived the following design goals:

- *G1* Tight integration of the geometric view and the attribute views to allow users a simultaneous visual analysis of both data facets.
- *G2* Support the localization of single and multiple cracks in the geometric view based on attribute criteria.
- *G3* Encode attribute values in the geometric view to allow users to judge their spatial distribution.
- *G4* Identification of clusters or outliers in the geometric and the attribute views.
- *G5* Incorporating exploration and visualization metaphors that users are already familiar with.

## Visualization and interaction design

In this design study, we combine a geometric view and three attribute views. The geometric view utilizes the 3D real-time rendering framework Aardvark [36], while the attribute views are part of the visual analysis tool Visplore [37]. We briefly cover basic coordinations between both parts and discuss attribute views with a closer look on the aggregation plot. The main focus of this design study is the integration-specific design decisions concerning the geometric view. We conclude the section with a similarity-based analysis as a suitable tool to explore geometric and attribute data.

### Linking and brushing, and color mapping

Linking and brushing is one of the core concepts of CMVs and allows users to identify tunnel cracks across views. In our implementation, brushing of cracks in one view results in a red highlighting of the selected cracks, which is linked with all other views (Fig. 3e). Peek brushing [1] causes a temporary selection of entities by hovering, which results in a blue highlighting (Fig. 3f). This allows users to instantly identify and compare tunnel cracks across all views. Users can map attribute values to colors, which are then shared among the views. If color mapping is active, we use size and transparency to emphasize selection and peek selection. Linking & brushing and color mapping are fundamental to meeting *G1* and *G3*, respectively.

### Attribute views

After implementing the aforementioned basic coordinations between the two tools, we discussed potential attribute views with our experts. We agreed on using a scatter plot (Fig. 3b), a parallel coordinates plot (PCP) (Fig. 3c), and an aggregation plot (Fig. 3d). Users found the *scatter plot* very intuitive, and suitable for detecting outliers and clusters with respect to two dimensions. For instance, Fig. 5a shows an outlier in a scatter plot of length and orientation, which is a mineral deposit as shown in Fig. 5c. Users were less familiar with the *PCP*, but welcomed that it offers an instant overview of all relevant data columns. They were especially fond of the possibility to specify arbitrary criteria by combining selections on multiple axes, as it is illustrated in Fig. 3e. The scatter plot and the PCP partly address *G4* since they allow users to identify and brush outliers and clusters.

The *aggregation plot* allows for splitting attribute data hierarchically along two dimensions. The individual parts of the data are presented as a matrix of counts or histograms. Together with our experts, we identified this view as suitable for estimating the density of cracks and identifying critical sections. To achieve this, we use the vertical dimension to distinguish between cracks in the south or in the north tunnel tube. We further map the Sv attribute to the horizontal axis and group it into intervals of 120 m, corresponding to the section size of the tunnel. This setup assigns cracks to individual tunnel tubes and tunnel sections. After mapping moisture values to the aggregation plot, each section shows individual counts of cracks for each of the three moisture values, as illustrated in Fig. 3d. Using the Sv coordinate as reference axis addresses *G5* and makes the aggregation plot very intuitive.

**Fig. 4:**
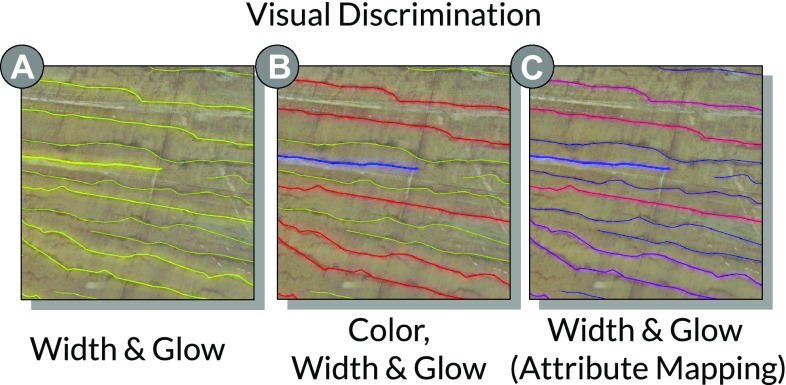
Visual discrimination: different visual variables are manipulated to emphasize the differences between peek-brushed, brushed, and context cracks. **a** Three different widths are used, while cracks in focus have a superimposed glow. **b** Using different widths and colors, *blue*, *red*, and *yellow-green* for peek brushed, brushed, and others. **c** Color is used for attribute mapping, while visual discrimination is only conveyed by width and glow.

### Geometric view

The geometric view provides an interactive rendering of the geometric representations of cracks and the tunnel surface. It allows users to interactively navigate the scene and to assess the spatial extent and distribution of the cracks. We visualize the tunnel cracks using a line shader with screen-space scaling, so each polyline maintains a certain pixel width, regardless of the distance to the viewer. Brushed and peek-brushed cracks (cracks in focus) are highlighted in red and blue (Fig. 4b), respectively, while the color of context cracks is yellow-green. To further ensure their visibility, brushed and peek-brushed cracks are rendered with a higher pixel width than context cracks. We further use a separated Gaussian blur filter to create a glow effect [10] (Fig. 4a), which we superimpose onto the cracks in focus. This also preserves visual discrimination of focus and context if color is used to encode attribute values (Fig. 4c).

**Fig. 5:**
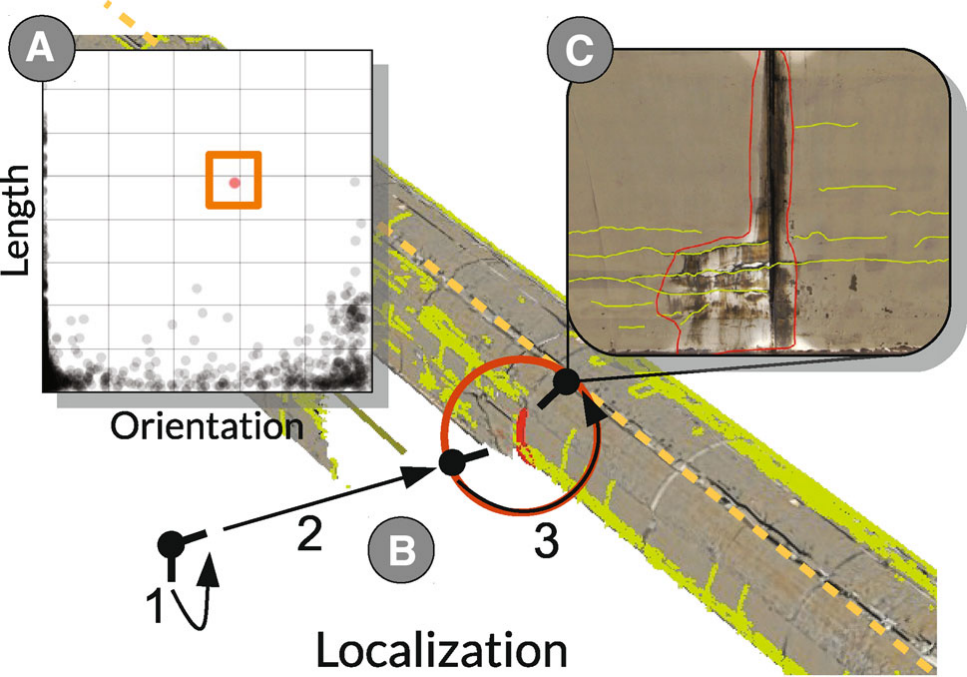
Localization transition: **a** Users brush a single crack in the scatter plot. **b** A localization transition is triggered (*1*) the camera turns to selected crack (*2*) the camera moves to closest point on a computed orbit sphere (*3*) the camera rotates around the selected crack and ends up the characteristic viewpoint inside the tunnel. **c** The scene rendered from characteristic viewpoint.

Our experts use the attribute views to get an overview of the multivariate part of their data. They either brush a single crack and seek to inspect its spatial representation (access), or they brush multiple cracks and are interested in their spatial distribution (spatial relation). In both cases, due to occlusion or cracks being outside of the current view frustum, this requires manual 3D navigation which is tedious and can lead to disorientation. To alleviate this and meet design goal *G2* we provide *guided navigation* techniques to ensure that all cracks in focus are inside the view frustum (Sect. 4.3.1). We use a *virtual X-ray technique* [8] and a *visual abstraction* [28] to counteract occlusion (Sect. 4.3.2).

#### Guided navigation

*Single crack* Together with our experts we defined what is a characteristic viewpoint [35] for a single crack, i.e., how should the 3D view provide access to a crack’s spatial representation. During a virtual tunnel inspection, analysts inspect individual cracks by ’standing’ on the tunnel axis (i.e., inside the tunnel surface and 1.70 m above the ground) and viewing them at an almost orthogonal angle, which is based on an actual on-site inspection (*G5*). Since each crack has an Sv coordinate, we can compute a corresponding position 1.70 m above the tunnel axis. Setting the look-at vector to the center of the crack results in the characteristic viewpoint for a single crack.

After users select a single crack in an attribute view (Fig. 5a), we employ an animated camera transition, the localization transition, illustrated in Fig. 5b: (1) we animate the camera’s look-at vector to focus on the brushed crack. Before translating, we compute a transitional viewpoint. We create a sphere centered on the crack, where the radius corresponds to the distance between the center of the sphere and the characteristic viewpoint. (2) We compute the closest point on said sphere as a transitional viewpoint and animate the camera position. (3) The camera is focused on the crack, orbits along the sphere, and reaches the characteristic viewpoint (Fig. 5c).

**Fig. 6:**
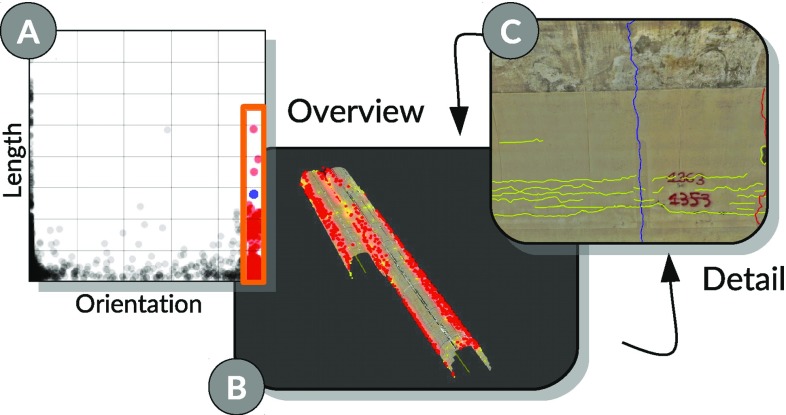
**a** Users investigate the distribution of cracks, which are orthogonal to the tunnel direction. **b** Selection triggers an automatic camera transition to a user-defined overview viewpoint. **c** Clicking on individual cracks seen from the overview viewpoint issues a localization transition, an additional click transitions back to the overview.

*Multiple cracks* The aggregation plot provides experts with exact distributions of attribute values with respect to tunnel sections. However, many tasks require analysts to judge the spatial distribution of attribute values more accurately and to gain immediate access to the corresponding spatial representations. Therefore, we employ camera transitions to allow users to intuitively investigate multiple cracks from overview and detail viewpoints.

After users select multiple cracks in an attribute view (Fig. 6a), which are typically distributed along the tunnel, we trigger an animated camera transition to a user-defined overview viewpoint (Fig. 6b). Right clicking on a crack from this position triggers a localization transition. An additional right-click transitions the camera back to the overview. This provides immediate access to detailed spatial representations, for instance, when identifying a cluster of moist cracks.

#### Handling occlusion and clutter

**Fig. 7:**
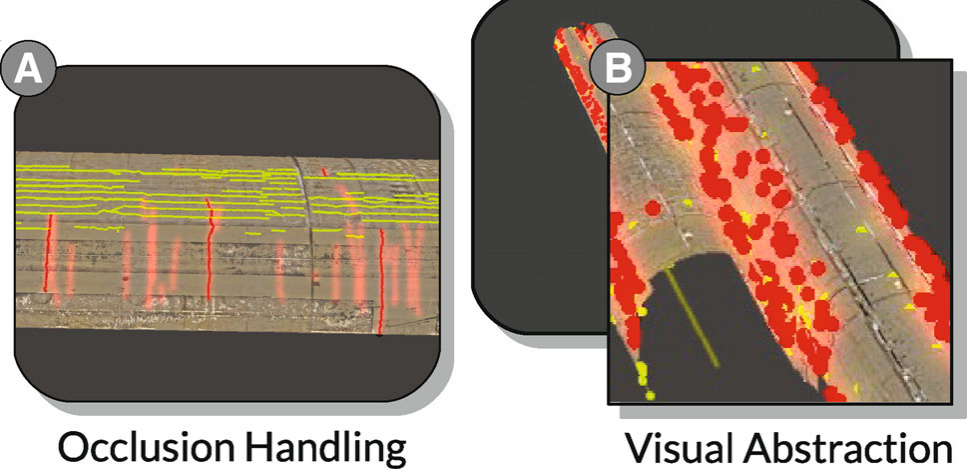
**a** Brushed cracks are highlighted in *red* including a glow effect. The glow effect persists even when the respective cracks are occluded. **b** Detailed tunnel crack representations are replaced by point sprites to avoid clutter.

*Virtual X-ray* Our localization transition moves the camera to a characteristic viewpoint that is free of occlusion. However, it might be confusing when users are guided to a crack that is occluded during most of the transition. Further, when inspecting the tunnel from an overview position, many cracks are occluded by the tunnel surface. Elmqvist and Tsigas [8] present a survey on 3D occlusion management techniques. Based on their categorization, we developed a virtual X-ray technique, which allows users to see cracks in focus through the tunnel geometry. We achieve this by rendering the aforementioned glow for each focus crack without depth testing. Consequently, as it is illustrated in Fig. 7a, the glows of the cracks in focus shine through the tunnel wall.

*Visual abstraction* In some sections of the tunnel, the cracks occur in a high density. Viewing these sections from an overview position, the display is easily cluttered and it becomes difficult for users to distinguish between individual cracks. To counteract this, we replace the polyline of a crack with a point sprite if a certain distance threshold is reached. This levels-of-detail approach, or more generally levels-of-abstraction [28], reduces visual clutter and allows users to identify individual crack positions (discovery) and their color (partial access). Consequently, using point sprites as visual abstractions also meets design goals *G3* and *G4*.

### Similarity-based analysis

In some scenarios, analysts want to compare entities with respect to a typical pattern of attribute values. For instance, if there is a dominant pattern of long, dry cracks, oriented along the tunnel direction, analysts are interested in cracks that deviate from this. Therefore, we provide a similarity-based analysis, which allows users to specify a point of interest in their data, i.e., the *focal point*. We quantify similarity by a *distance metric* and treat the resulting distance as another attribute value for each crack. Color mapping enables the identification of tunnel cracks that are similar to or deviate from the specified focal point, which serves *G4*.

Focal points are typically used in the context of parameter-space exploration [2, 24]. In general, a focal point is a user-defined *n*-tuple specifying concrete values for all or a subset of the *n* attributes of a data entity. Further, Berger et al. [2] discriminate *global* and *local* updates of a focal point. Local updates only affect a subset of the attributes, while global updates affect all attributes at once.

For the distance computation, we use a normalized Euclidean distance metric between a crack and the focal point with equally weighted components. Users can select the attributes they want to incorporate into the similarity computation. The focal point can be locally updated by specifying values on the axes of the scatter plot or the axes of the PCP. The respective coordinates of the focal point are represented by green lines. In the geometric view, users can perform a global update by selecting a crack as the focal point resulting in a green highlight.

### Expert feedback

We conducted informal feedback sessions for confirming the usefulness of the developed tunnel-crack analysis tool. We interviewed four domain experts: two of whom are from the field of tunnel maintenance (A) and monitoring (B), whereas the others are from the fields of urban planning (C), and disaster management (D). Experts A and B were already familiar with using a 3D tunnel visualization for exploration of geometric data. Since multivariate analysis is mostly conducted in a paper-based, non-interactive form, they were eager to specify arbitrary selection criteria in the PCP and the scatter plot and successively refined them.

When investigating multiple cracks, experts A and B found the overview and detail transitions very helpful. All experts found the localization of focus cracks and the localization transition essential for exploring the geometric view based on attribute criteria. Further, all experts deemed the overview viewpoint and the visual abstractions valuable, since many of their tasks involve the assessment of spatial distribution. Expert C explicitly complimented the implementation of user-specified overview viewpoints. He suggested to add a list for the management of multiple overview viewpoints.

Experts A and B, found the glow implementation for occlusion management helpful for orientation, but found it confusing during virtual inspection. Consequently, we deactivate the effect when the camera is inside the tunnel. Expert C and D stated that the effect would be useful in an urban scenario, for instance, when objects are hidden behind buildings. When analyzing cracks in a detail viewpoint, two of the four experts desired an orbit navigation mode in addition to the implemented free-fly camera movement.

After some explanation, the experts C and D could see the potential of similarity-based analysis, but they could not immediately imagine how this would translate to their use cases. The tunnel experts A and B on the other hand could immediately grasp the benefit of similarity-based analysis, when applied to the tunnel maintenance scenario. Expert B stated that similarity-based analysis would also translate very well to a tunnel monitoring use case. During construction of the tunnel, segments of it are allowed to shift within a given range of horizontal and vertical movement depending on the type of rock, which surrounds the segment. Considering a large number of shifting measurements along the tunnel over time, it would be helpful to explore them by dissimilarity to normative values. Further, it would be interesting to add time-dependent data from deviation measurements and surface geometry of the growing tunnel. In general, our integrated solution was well received. All experts could see the value of a system effectively combining geometric and attribute views on their data. The tunnel experts saw it as a solution to support currently cumbersome tasks.

## Implementation

We chose to combine two existing systems, the visual analysis tool Visplore [37] and the 3D real-time rendering engine Aardvark [36]. Visplore is designed to handle and visualize large amounts of multivariate tabular data. Besides typical 3D rendering features, Aardvark can import various data formats and manages out-of-core streaming of large geometry and texture data. Both systems are trimmed to scale well with their respective data facet and were able to render the scenario at interactive frame rates. Both systems are used separately for numerous application-oriented research projects, such as urban planning, tunnel documentation, engine design, and power management, and are not publicly available. We extended both systems with a custom-built communication layer based on web sockets, which allowed us to handle the coordination between the two systems by exchanging JSON messages.

## The VISAR framework

As outlined in Sect. 2, many authors combine geometric and attribute views through similar integration approaches. Their discussion, however, is often focused on the particular application rather than on general applicability. Based on the insights gained during conduction of the presented design study and based on the review of related literature, we could identify common obstacles and recurring design questions inherent to the integration of geometric and attribute data. As a result, we present VISAR, a methodological framework addressing this integration on a more general level to support visualization designers in effectively combining geometric and attribute views.

Integrated solutions, which are tailored to a specific use case, typically focus on the analysis of a certain data entity: flooded buildings in disaster management [25], buildings [11] and lines-of-sight [22] in urban planning, illuminated surfaces [30] in interior lighting design, or tunnel cracks in the presented scenario. We generalize these entities as $e = (g,a)$, where *g* is the geometric spatial representation and $a=(a_{1},\ldots ,a_{n})$ is the attribute vector of length *n*. Analogous to the tunnel cracks, entities of the form *e* are subject to the problem space described in Sect. 3.3, since *g* may lie outside the current view frustum or may be occluded by other geometry in the scene. Although concrete tasks and actual design decisions depend on the specific use case, it is essential that an integrated approach allows the accomplishment of all visual perception tasks.

**Fig. 8:**
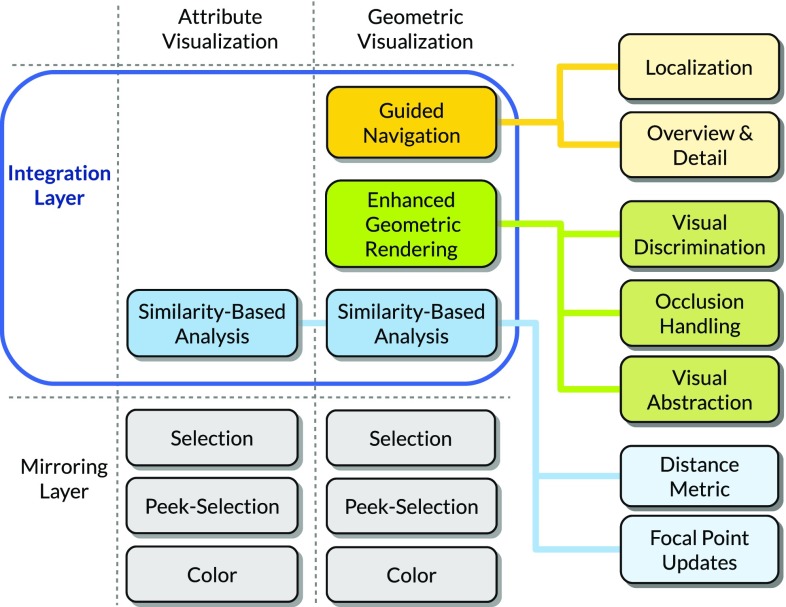
The VISAR framework is divided into two layers: the mirroring layer and the integration layer. The mirroring layer contains simple coordinations, such as Selection, Peek Selection, and Color. The components of the integration layer are concerned with more complex coordinations that facilitate the visual perception tasks (see Sect. 3.3): Guided Navigation, Visual Encoding, and Similarity-Based Analysis.

To support visualization designers in achieving this goal, our methodological framework addresses the integration between heterogeneous systems on two levels, which are reflected in the two-layer structure of VISAR: the *mirroring layer* and the *integration layer* (Fig. 8). The mirroring layer covers coordinations between views in general, while the integration layer explicitly deals with the coordination of geometric and attribute views to prevent visual perception tasks from failing.

### Mirroring layer and integration layer

The *mirroring layer* is responsible for straightforward coordinations, which are shared among all views regardless of their type. This includes the selection, i.e., brushing, of entities or the encoding of attribute values into entity colors. Since the mirroring layer encompasses principles implemented in most CMV systems, it acts as the foundation of the actual integration. This includes coordinated interactions, such as linking & brushing [12], focus & context [17, 34], or coordinated color mapping [13, 16].

The *integration layer* is concerned with the coordination between geometric and attribute views and its components explicitly address the discussed problem space to facilitate the visual perception tasks. Through literature review and generalization of the implementations discussed in Sect. 4, we derived the components *guided navigation*, *enhanced geometric rendering*, and *similarity-based analysis* for the integration layer. We will elaborate on each component and its sub-components adhering to the following structure: purpose of the component, design goals of its subcomponents, design choices, and comparison to the literature.

### Guided navigation

The purpose of the guided navigation component is to allow users the localization of the geometric representations of entities in focus by means of automated camera transitions. The intent of localization is typically preceded by brushing one or more entities in an attribute view. We distinguish two goals:

1. Provide users with the discovery and detailed shape access of a single entity.
2. Provide users with the discovery of multiple entities and allow users to judge their spatial relation.

*Localization* We address (1) by a localization transition that animates the camera to a characteristic viewpoint. The transition itself and the characteristic viewpoint typically depend on the use case. Design choices are the computation of a characteristic viewpoint and the definition of a transition path that does not cause disorientation. Viola et al. [35] use an information theory approach to estimate characteristic viewpoints of internal organs in a medical data set. When selecting an organ a localization transition is triggered and moves the camera along a bounding sphere. Buchholz et al. [4] discuss a constraint-based framework for navigation in virtual 3D landscapes.

*Overview and detail* We address (2) by an overview transition that animates the camera to reach an overview viewpoint, which contains all entities in focus. Design choices concern the definition of a suitable overview viewpoint and how to provide a transition that prevents users from disorientation. Although there is no example actually computing a characteristic overview viewpoint for a changing set of focus entities, approaches can be found in the category of tour planning techniques in Elmqvist and Tsigas [8].

### Enhanced geometric rendering

The enhanced geometric rendering component manipulates the geometric representations of entities to achieve the following goals:

1. Discrimination between entities in focus and entities or other geometric objects as part of the context.
2. Completing all visual perception tasks for entities in focus despite suffering from occlusion.
3. Allowing users to judge spatial relation and provide access to encoded values from overview viewpoints.

*Visual discrimination* In general, (1) is achieved by highlighting geometry in focus and/or lowlighting geometry belonging to the context [14]. In many cases, the highlighting / lowlighting of the geometric representation of an entity requires design decisions that preserve color, textures, size, or shape, which may be essential to an effective analysis. Trapp et al. [34] evaluated several techniques for highlighting in 3D geovirtual environments. Outline-based techniques and style-variant techniques, that consider use-case context, are suited best to enable visual discrimination and preserve relevant spatial properties.

*Occlusion management* To address (2), a suitable occlusion management technique is necessary to ensure that discovery, access, and spatial relation tasks do not fail, although the entities in focus are occluded. Elmqvist and Tsigas [8] provide a wide variety of design alternatives. Virtual X-ray techniques appear to be most suitable for geometric views combined with attribute views, since they are reliable and preserve the most geometric properties.

*Visual abstraction* With increasing distance, and also dependent on the density entities occur in, the display becomes cluttered and discovery is impeded. We address (3) by replacing the entities by visual abstractions. Such an abstraction may range from a geometric simplification, to a glyph representations summarizing multiple entities [22], up to small attribute views integrated into the 3D scenes  [5, 30]. Chang et al. [7] employ a hierarchical simplification of city models adhering to perception constraints derived from urban planning [6]. Semmo et al. [28] developed a levels-of-abstraction framework for blending between discrete simplification levels for various geospatial features.

### Similarity-based analysis

Similarity-based analysis allows users to explore their data by means of similarity and deviation with the following goals:

1. Comparison of entities with respect to a user-specified focal point in the data.
2. Detecting clusters of similar entities or identification of entities deviating from a common pattern.

*Similarity* We define the similarity of an entity *e* to the focal point *f* as follows:

1 $$\begin{aligned} {\text{ s }imilarity}(f,e) = \left( \displaystyle \sum \limits _{i=1}^n w_i \cdot {\text{ d }ist}\left( f_i, e_i\right) ^p\right) ^\frac{1}{p} \end{aligned}$$

For dist(*f*, *e*) different distance metrics can be inserted, such as the Euclidean distance ($p=2$). Further, different weights can be applied in form of the *n*-dimensional weighting vector *w*. Which distance metric to use depends on the application scenario and the involved data types. Migut et al. [19] present approaches for metrics considering nominal attributes and weights. Mesh comparison metrics, as presented by Schmidt et al. [27], allow the integration of the geometric part of the data into the similarity computation.

*Focal point updates* Berger et al. [2] distinguish *global* and *local* updates of the focal point. An integrated solution may allow local or global updates of the focal point in geometric or attribute views. Selecting a particular entity as the focal point in any view is a straightforward way for a global update. In Legible Cities [7], users can select individual neighborhoods as focal point in 3D by a pin metaphor in a census dataset.

## Discussion

We developed an effective solution for the visual analysis of tunnel cracks by integrating geometric and attribute views. We derived VISAR, which we see as an extended discussion of our design study in terms of generalization and believe in its applicability to a wide range of use cases involving geometric and attribute data. At this point we want to briefly reflect on the limitations and possible extensions of this methodology.

The VISAR methodology mostly concerns the side of the geometric visualization, which is based on the fact that the three visual perception tasks are more likely to fail in 3D space than in the visualization space of the attribute visualizations. Further, our users tended to employ attribute views for exploring the geometric view, which is denoted as explore & feedback [31]. However, we realized, that the visual feedback of, for instance, scatter plots reacting to a selection in the geometric view is often not sufficient to direct a users’s visual attention. This issue could be alleviated using visual links [38] connecting geometric representations and attribute representations. In general, our methodology strives to generate solutions employing a balanced integration to allow a continuous feedback loop between both visualization domains [31].

## Conclusion and future work

In this paper, we present an integrated solution, as the result of a design study, to support experts in tunnel crack analysis in the context of a tunnel maintenance scenario. We evaluated the resulting visual analysis tool with our experts and gained overall very positive feedback. We further derived VISAR, a methodological framework to assist visualization designers which building integrated solutions in similar heterogeneous scenarios. We plan to use VISAR in future projects, as for instance, in an extension of the tunnel maintenance scenario which will encompass tunnel deformation data over time, or in a project concerned with remote geological analysis on Mars involving derivation and interpretation of statistical data from thousands of geological measurements. While employing VISAR ourselves, we will promote it to fellow research groups to be able to gather valuable feedback concerning VISAR’s true applicability and usefulness on a broader scope.

## Electronic supplementary material

Below is the link to the electronic supplementary material.

Supplementary material 1 (mp4 25550 KB)
